# Synthetic anthocyanidins and their antioxidant properties

**DOI:** 10.1186/s40064-015-1250-x

**Published:** 2015-09-17

**Authors:** Homar S. Barcena, Peishan Chen, Abraham Tuachi

**Affiliations:** Physical Sciences, Kingsborough Community College, Brooklyn, NY 11235 USA

**Keywords:** Dyes, Green chemistry, FRAP, One pot synthesis

## Abstract

Anthocyanidins were synthesized to study the effect of methoxy substitution on the B ring to their antioxidant property. Comparative FRAP studies show 2′- and 4′-methoxy substituents have higher antioxidant activities, which may be attributed to both resonance and inductive effects.

**Electronic supplementary material:**

The online version of this article (doi:10.1186/s40064-015-1250-x) contains supplementary material, which is available to authorized users.

**Graphical abstract:**
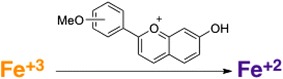
Anthocyanidins as reducing agents

## Background

Anthocyanidins are pigments that are associated with the bright coloration of flowers and fruits. These natural dyes belong to the flavonoid family, with their basic structure comprising of an aromatic ring (A) fused with an heterocyclic ring containing an oxygen (C), which is also bonded to a third aromatic ring (B). These compounds are normally substituted with hydroxy groups, which help stabilize the charge on the flavylium cation. When one of the phenols is substituted with glycosides, the compound is called an anthocyanin.

The natural occurrence of anthocyanins and anthocyanidins warrants their study not only for the evolutionary advantage they confer to plants, but also for their potential applications (Castañeda-Ovando et al. 2009). Besides their utility as colorants for foods and cosmetics (Campanella et al. 2010), they are also explored in materials science (Pina et al. 2012) for example, as photosensitizers for photovoltaics (Calogero et al. 2013; Gokilamani et al. 2013), and as molecular logic gates (Pina et al. 1998). Like many polyphenols, they exhibit biological activities that are beneficial to human health (Pojer et al. 2013) such as in glucose metabolism (Alzaid et al. 2013), protection against cardiovascular disease (Wallace 2011), and mediation of oxidative stress (Zafra-Stone et al. 2007). Their putative roles in human pathologies are of interest, particularly in cancer prevention (Wang and Stoner 2008; Cooke et al. 2005). Despite their biological significance, their pharmacokinetics in humans remains largely unexplored (Kay 2006). Thus, to further the utility of anthocyanins in therapeutics and gain an understanding of their activities as applied to drug design, we synthesized anthocyanidins **1**–**3** and studied their antioxidant properties.

There are several methods for determining and expressing antioxidant activity, particularly for natural anthocyanins extracted from plants (Thaipong et al. 2006; Huang et al. 2005; Pulido et al. 2000; Sochor et al. 2010). This paper reports the preparation and characterization of three new anthocyanidins with different substitution patterns on the B ring. The antioxidant activities of the synthetic anthocyanidins were studied using a modified ferric reducing activity of plasma (FRAP) assay (Benzie and Strain 1996, 1999).

## Results and discussion

Synthesis of the flavylium cation occurs under harsh conditions (Balaban et al. 1969) and preparations of anthocyanidins have been achieved by bubbling the reaction with hydrogen chloride gas (Moncada et al. 2004), treatment with perchloric acid (Sato et al. 1999; Dorofeenko and Olekhnovich 1972), or employment of corrosive Lewis acids such as boron trifluoride etherate (Kuhnert et al. 2001). Recently, milder synthesis using sulfuric acid was reported (Calogero et al. 2013), and described herein is a convenient approach to obtaining anthocyanidins, using less solvent and shorter reaction times. A summary of synthetic methods is listed in Table 1 and the synthesis of flavylium ring has been comprehensively reviewed elsewhere (Iacobucci and Sweeny 1983).

**Table 1:** Reported syntheses of anthocyanidins

Conditions	Yield (%)	References
Salicylaldehyde, acetophenone, HBF_4_, HOAc, acetic anhydride, 60 °C, 12 h	40–58, 23–78	Katritzky et al. (1998), Gomes et al. (2009)
Salicylaldehyde, acetophenone, BF_3_ etherate, neat	81	Kuhnert et al. (2001)
Salicylaldehyde, acetophenone, H_2_SO_4_, HOAc, overnight	40–88	Calogero et al. (2013)
Salicylaldehyde, acetophenone, EtOAc, HCl gas, 0 °C, 3 days	56–75, 55–84	Mora-Soumille et al. (2013), Mas (2003)
Salicylaldehyde, acetophenone, HPF_6_, HOAc, 2 days	89	Kueny-Stotz et al. (2008)
Salicylaldehyde, acetophenone, HCl gas, formic acid, 5 h	56	Moncada et al. (2004), Michaelidis and Wizinger (1951)
Salicylaldehyde, benzaldehyde, ethyl chloroformate, HClO_4_, 1–12 h	49–95	Sato et al. (1999)
Salicylideneacetophenone, HBF_4_OEt_2_ or HOTf in Et_2_O	62–67	Fichtner et al. (2001)
Phenol, arylethynylketone, HPF_6_, HOAc, r.t.	82–99	Kueny-Stotz et al. (2007)

**Scheme 1:**
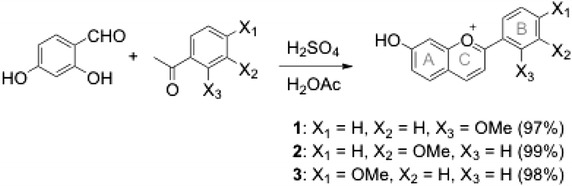
Synthesis of anthocyanidins **1**–**3**

Scheme 1 shows the condensation of 2,4-dihydroxybenzaldehyde with different acetophenone derivatives using a minimum amount of acetic and sulfuric acid. Heating in a water bath for 30 min facilitated the reaction, which resulted in a dark viscous liquid. The products were purified by trituration with diethyl ether. When performed with minimum exposure to air, fine, brightly colored powders are obtained, which were dried further in a vacuum desiccator. The hygroscopic anthocyanidins were assumed to be bisulfate salts, and the yields were 92–95 %. While the use of concentrated sulfuric acid is still harsh, improvements such as shorter heating time, use of the renewable solvent acetic acid, minimum solvents and adjuvants used during purification, and high yields makes our procedure greener. Characterization by ^1^H and ^13^C NMR and HRMS confirms the products, which have nearly similar UV–Vis and IR spectra in the functional group region.

Solutions of **1**–**3** were prepared by first dissolving in DMF, and subsequent dilution with acetate buffer (pH 3.6). Flavylium salts are in equilibrium with their hydrates in aqueous solutions, with low pH favoring the non-hydrated pyrilium cation (Moncada et al. 2004). Once hydrated, they may undergo ring opening, then tautomerization to the enone, and further isomerization to give *trans* chalcones. Buffered solutions of **1**–**3** showed no variation in the UV spectra when kept in the dark, and when kept cold for at least 1 week, hinting on their stability.

**Fig. 1:**
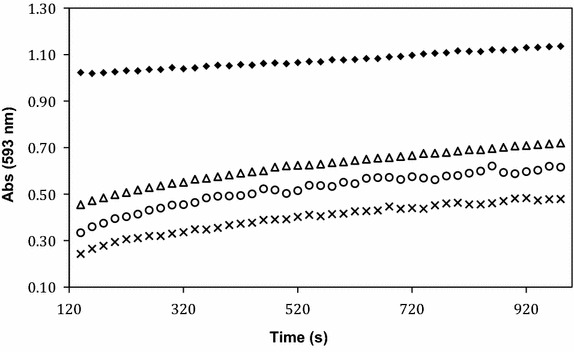
FRAP assay of synthetic anthocyanidin **1** (*open triangle*), **2** (*multiplication sign*), and **3** (*open circle*). Vitamin C (*filled diamond*) shows higher antioxidant activity under similar conditions. The antioxidants are of the same final concentration (0.15 mM), and the final concentration of Fe^3+^(TPTZ)_2_ was 735 mM

A modified FRAP assay was used to study the antioxidant properties of **1**–**3**. Freshly prepared FRAP reagent was admixed with antioxidants at room temperature, which showed rapid development of color characteristic of the formation of the Fe^2+^ complex. Spectrophotometric measurements were taken 2 min after mixing and all studies were performed in triplicate. The initial color change was fast, however the redox reaction continued for longer than 15 min, similar to what has been observed in polyphenol antioxidants (Pulido et al. 2000). Varying the location of the methoxy substituent on the C ring offers slight differences in the reducing power of the synthesized flavylium salt, with **1** showing the highest antioxidant activity (Fig. 1). This may be attributed to the added stability conferred by conjugation with the B ring substituents (Calogero et al. 2013). It can be reasoned that the higher activity of **1** compared to **3** is due to inductive effects of the proximal 2′ methoxy to the flavylium oxygen, which is absent in the 4′ methoxy (see Additional files 1, 2). The resonance effect is absent for the 3′ methoxy, resulting in least stable derivative (**2**).

The solution chemistry of anthocyanidins is complex (Pina et al. 2012) and analogous anthocyanidins under similar pH exist in equilibrium between the flavylium ion, deporotonated quinoidal base, and as the hydrated hemiketal (Brouillard et al. 1982; Sweeny and Iacobucci 1983). The FRAP assay is non-specific for any antioxidant present under the reaction conditions that could reduce Fe^3+^, which takes into account the chemistry flavylium ions undergo in solution. Under similar assay conditions, ascorbic acid gives higher FRAP value (2.7) and shows a higher antioxidant activity than anthocyanidins **1**–**3**. FRAP values are normally obtained after 4 min at 37 °C, or 6 min at room temperature. No significant variation of the FRAP value was observed between 4 and 6 min for our experiments, which are 2.2, 2.0, and 2.1 mM for **1**, **2**, **3**, respectively, based on equivalent FeSO_4_ standard. In comparison, purified anthocyanin extracts from fruit show reducing power one-third that of ascorbic acid, however these comparisons are not straightforward because the reducing power is dose-dependent even for ascorbic acid (Sun et al. 2014).

## Conclusion

In conclusion, we demonstrate a greener synthesis of anthocyanidins, which allows facile purification by trituration. This facilitates the study of the effects of various substituents on the different rings to the properties of anthocyanidins. In this case, we show that altering the location of the methoxy substituent on the B ring results in slight variations in the resultant antioxidant activity, as measured by the FRAP assay. The methoxy substituent on the 2′ position of the B ring stabilizes the radical formed in the 7-OH position by conjugation, and by inductive effects due to the proximity of the the methoxy group to the pyrilium oxygen. These results demonstrate the feasibility of tailoring the redox properties of synthetic anthocyanidins.

## Experimental

All starting materials and solvents were purchased from commercial sources. NMR analyses were performed using a Bruker 400 MHz Avance, and IR analyses were performed using a Bruker Alpha ATR-IR. High-resolution mass spec were obtained from The City College of New York Mass Spectrometry Facility, and the counter anion was not included in the molecular ion peak calculations.

### General procedure for FRAP

Freshly prepared FRAP solution was prepared by mixing acetate buffer at pH 3.6 (10.0 cm^3^, 20 mM), TPTZ solution (1.0 cm^3^, 10 mM), and FeCl_3_ solution (1.0 cm^3^, 10 mM) in a vial. Stock solutions of the anthocyanidins (35.0 mg) were prepared in DMSO (100 cm^3^, 1 mM). All solutions were sparged with N_2_ prior to each experiment. For each experiment, the stock was diluted to 0.5 mM with acetate buffer and equilibrated for 3 min. The experiment was initiated in a new vial containing de-ionized water (900 μL) and TPTZ solution (9.0 cm^3^). To this was added the diluted anthocyanidins (300 μL), mixed, and immediately transferred to a cuvette. Data capture was started exactly 2 min after the reaction was initiated. The blank was prepared similarly, but adding only buffer instead of the stock anthocyanidin solution. Each experiment was repeated at least three times.

### General procedure for anthocyanidins

To a 25-cm^3^ round bottomed flask was added 2,4-dihydroxybenzaldehyde (414 mg, 3.00 mmol) and the corresponding methoxyacetophenone isomer (0.413 cm^3^, 3.00 mmol). The mixture was dissolved in acetic acid (1.00 cm^3^), and sulfuric acid (0.500 cm^3^) was added. The mixture was equipped with an air condenser and heated in a boiling water bath for 30 min. The solid product was obtained by triturating the oil with diethyl ether (2.0 cm^3^). Purification was achieved by dissolving the crude in acetic acid and triturating with ether at least three times. The product was vacuum filtered and washed with diethyl ether before drying in a vacuum desiccator.

#### 7-hydroxy-2-(2-methoxyphenyl)chromenylium hydrogen sulfate (**1**, C_16_H_14_O_7_S)

Rust-colored powder, 0.994 g (95 %). M.p.: 100–107 °C (decomposed); ^1^H NMR (400 MHz, MeOH-d_4_) δ = 9.2 (d, 1H, *J* = 8.7 Hz), δ = 8.7 (d, 2H, *J* = 8.7 Hz), δ = 8.4 (dd, 1H, *J* = 8.08, 1.6 Hz), δ = 8.2 (d, 1H, *J* = 9.0 Hz), δ = 7.8 (td, 1H, *J* = 7.9, 1.6 Hz), δ = 7.51 (d, 1H, *J* = 1.6 Hz), δ = 7.48 (dd, 1H, *J* = 9.0, 2.2 Hz), δ = 7.38 (d, 1H, *J* = 8.6 Hz), δ = 7.3 (m, 1H), δ 4.1 (s, 3H); ^13^C NMR (100 MHz, MeOH-d_4_) δ 170.3, 170.0. 161.1, 160.3, 154.5, 137.8, 132.8, 131.0, 122.1, 121.7, 119.9, 117.8, 117.0, 113.0, 102.1 55.6 ppm; HRMS (ESI) *m/z* 253.0897 (M^+^), calcd for C_16_H_13_O_3_ 253.0865.

#### 7-hydroxy-2-(3-methoxyphenyl)chromenylium hydrogensulfate (**2**, C_16_H_14_O_7_S)

Dark red powder, 0.966 g (92 %). M.p.: 122–155 °C (decomposed); ^1^H NMR (400 MHz, MeOH-d_4_) δ = 9.3 (d, 1H, *J* = 8.5 Hz), δ = 8.5 (d, 1H, *J* = 8.5 Hz), δ = 8.3 (d, 1H, *J* = 9.0 Hz), δ = 8.1 (d, 1H, *J* = 8.2 Hz), δ = 8.0 (s, 1H), δ = 7.64 (m, 1H), δ = 7.62 (d, 1H, *J* = 1.8 Hz), δ = 7.5 (dd, 1H, *J* = 9.0, 2.1 Hz), δ = 7.4 (dd, 1H, *J* = 8.3, 1.9 Hz), δ = 4.0 (s, 3H); ^13^C NMR (100 MHz, MeOH-d_4_) δ 171.9, 170.6, 160.9, 160.2, 155.1, 133.1, 131.0, 130.5, 122.5, 121.8, 121.4, 120.5, 113.4, 113.1, 102.3, 55.0 ppm; HRMS (ESI) *m/z* 253.0890 (M^+^)^+^), calcd for C_16_H_13_O_3_ 253.0865.

#### 7-hydroxy-2-(4-methoxyphenyl)chromenylium hydrogensulfate (**3**, C_16_H_14_O_7_S)

Orange-red powder, 0.990 g (95 %). M.p.: 157–190 °C (decomposed); ^1^H NMR (400 MHz, MeOH-d_4_) δ = 9.1 (d, 1H, *J* = 8.7 Hz), δ = 8.5 (d, 2H, *J* = 9.1 Hz), δ = 8.4 (d, 1H, *J* = 8.7 Hz), δ = 8.2 (d, 1H, *J* = 9.0 Hz), δ = 7.5 (d, 1H, *J* = 2.0 Hz), δ = 7.4 (dd, 1H, *J* = 8.9, 2.2 Hz), δ = 7.3 (d, 2H, *J* = 9.1 Hz), δ = 4.0 (s, 3H); ^13^C NMR (100 MHz, MeOH-d_4_) δ 173.7, 170.8, 168.7, 160.1 155.2, 143.2, 133.6, 122.83, 122.76, 120.6, 117.2, 113.7, 103.8, 57.0 ppm; HRMS (ESI) *m/z* 253.0889 (M^+^), calcd for C_16_H_13_O_3_ 253.0865.

## Additional files

**Additional file 1.** Checklist for compound characterization.
**Additional file 2.** Compound characterization data.

## Abbreviation

FRAP: ferric reducing activity of plasma
