# Supplementary material for: Synthetic anthocyanidins and their antioxidant properties
Source: Springerplus. 2015 Sep 17;4:499. doi: 10.1186/s40064-015-1250-x (PMC4573978; doi:10.1186/s40064-015-1250-x)
Supplement: Supplementary file 1 — Additional file 1. Checklist for compound characterization. [file 40064_2015_1250_MOESM1_ESM.docx]

| **Compound** | | | | **New compounds** | | | | | | | | **Known compounds** | | |
| --- | --- | --- | --- | --- | --- | --- | --- | --- | --- | --- | --- | --- | --- | --- |
| Compound number | Systematic name given | New | Known | ^1^H NMR | ^13^C NMR | IR | MS | UV-Vis | Melting point | R_f_ value | Elemental analysis | Melting point | Reference to melting point in literature | Reference to NMR data in literature |
| 1 | x | x |  | x | x | x | x | x | dec. 105-107 |  | HRMS |  |  |  |
| 2 | x | x |  | x | x | x | x | x | dec. 122-155 |  | HRMS |  |  |  |
| 3 | x | x |  | x | x | x | x | x | dec. 157-190 |  | HRMS |  |  |  |

Additional file 1: Compound Characterization Checklist
