# Supplementary material for: Synthetic anthocyanidins and their antioxidant properties
Source: Springerplus. 2015 Sep 17;4:499. doi: 10.1186/s40064-015-1250-x (PMC4573978; doi:10.1186/s40064-015-1250-x)
Supplement: Supplementary file 2 — Additional file 2. Compound characterization data. [file 40064_2015_1250_MOESM2_ESM.docx]

**Additional File 2: Compound Characterization Data**

**Synthetic Anthocyanidins and their Antioxidant Properties**

Homar S. Barcena,^*^ Peishan Chen, and Abraham Tuachi

Kingsborough Community College, 2001 Oriental Blvd, Brooklyn, NY 11235

**Table of Contents**

Figure S1 2

Figure S2 2

Figure S3 ^1^H NMR of **1** 3

Figure S4 ^13^C NMR of **1** 4

Figure S5 UV-Vis of **1** 4

Figure S6 ATR-IR of **1** 5

Figure S7 HRMS of **1** 5

Figure S8 ^1^H NMR of **2** 6

Figure S9 ^13^C NMR of **2** 7

Figure S10 UV-Vis of **2** 7

Figure S11 ATR-IR of **2** 8

Figure S12 HRMS of **2** 8

Figure S13 ^1^H NMR of **3** 9

Figure S14 ^13^C NMR of **3** 10

Figure S15 UV-Vis of **3** 10

Figure S16 ATR-IR of **3** 11

Figure S17 HRMS of **3** 11

**Figure S1.** Methoxy substituent on the 2’ position of the B ring in **1** stabilizes the radical formed in the 7-OH position by conjugation, and by inductive effects due to the proximity of the methoxy group to the pyrilium oxygen.

**Figure S2.** The methoxy substituent on the 4’ position of the B ring in **3** stabilizes the radical formed in the 7-OH position by conjugation.


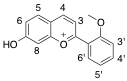

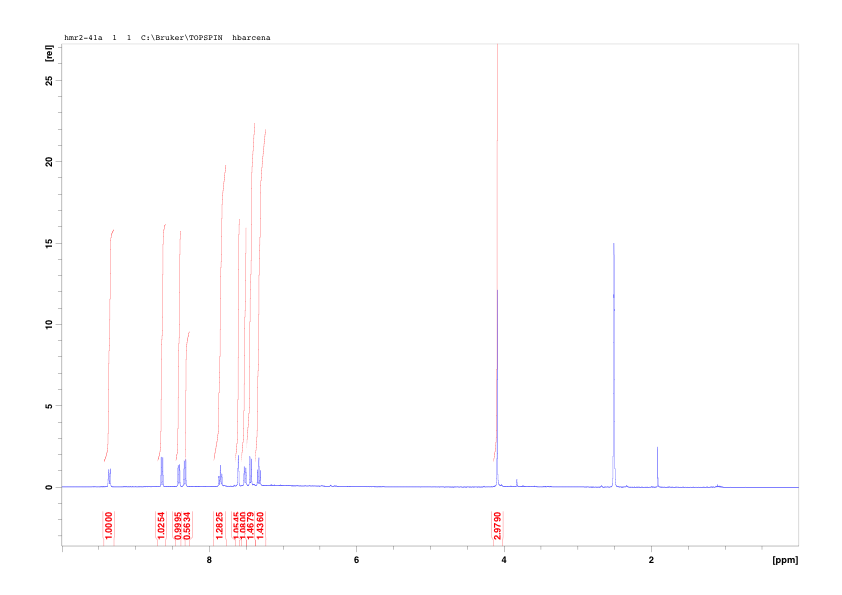


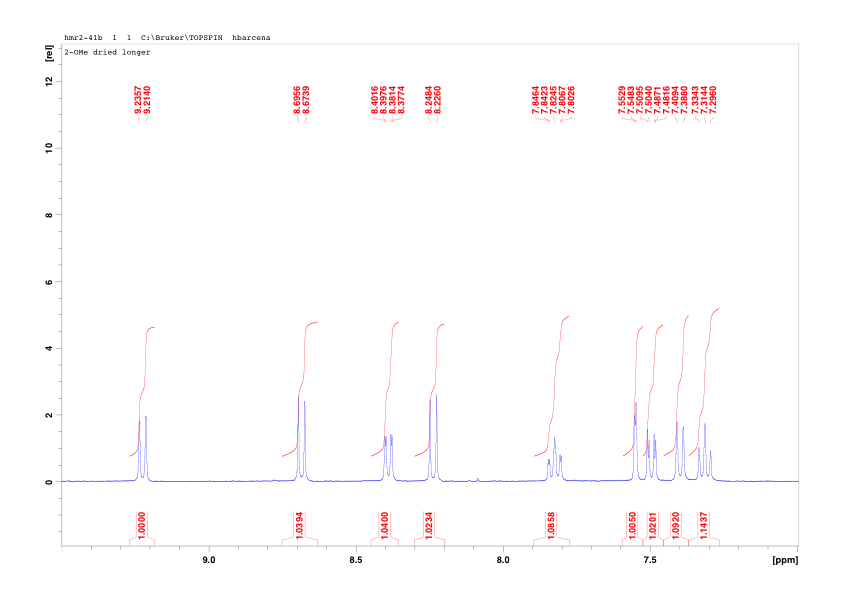


4 3 6 8 5

**Figure S3**. ^1^H NMR of **1**, MeOH-d4 400 MHz


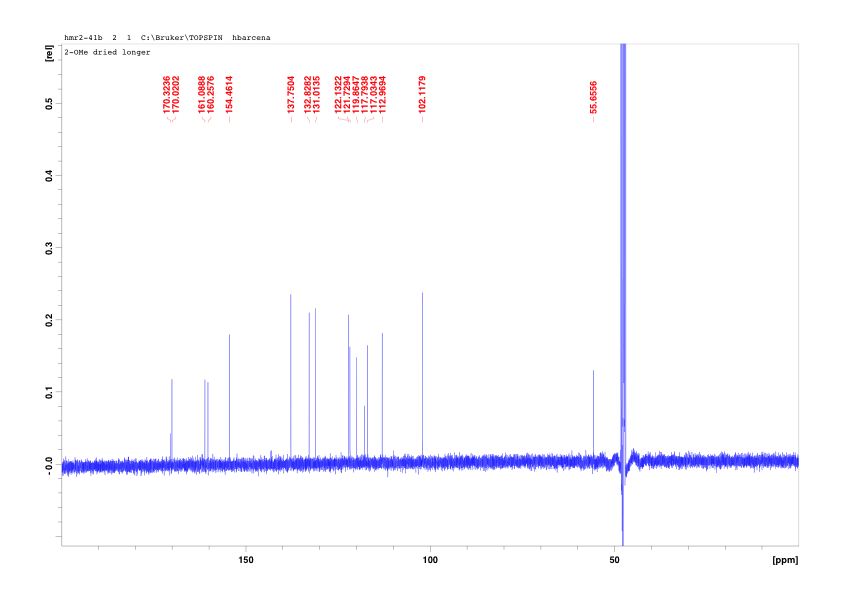


**Figure S4**. ^1^H NMR of **1**, MeOH-d4 100 MHz

**
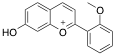
**

**Figure S5**. UV-Vis of **1** in acetate buffer (pH 3.6), λ_max_ = 365 nm, ε = 44,869 M^-1^cm^-1^

**Figure S6**. ATR-IR of **1**

**Figure S7**. HRMS of **1**

4 3 6’ 6 2’ 4’ 8 5’ 5

**Figure S8**. ^1^H NMR of **2**, MeOH-d4 400 MHz

**Figure S9**. ^13^C NMR of **1**, MeOH-d4 100 MHz


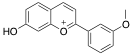


**Figure S10**. UV-Vis of **2** in acetate buffer (pH 3.6), λ_max_ = 373 nm, ε = 49,064 M^-1^cm^-1^

**Figure S11**. ATR-IR of **2**


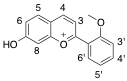


**Figure S12**. HRMS of **2**

4 3’ 3 5 8 6 2’

**Figure S13**. ^1^H NMR of **3**, MeOH-d4 400 MHz

**Figure S14**. ^13^C NMR of **3**, MeOH-d4 100 MHz

**Figure S15**. UV-Vis of **3** in acetate buffer (pH 3.6), λ_max_ = 372 nm, ε = 64,254 M^-1^cm^-1^

**Figure S16**. ATR-IR of **3**

**Figure S17**. HRMS of **3**
